# Depressive inclinations mediate the association between personality (neuroticism/conscientiousness) and TikTok Use Disorder tendencies

**DOI:** 10.1186/s40359-024-01541-y

**Published:** 2024-02-17

**Authors:** Christian Montag, Sebastian Markett

**Affiliations:** 1 Department of Molecular Psychology, Institute of Psychology and Education, Ulm University, Helmholtzstr. 8/1, Ulm, 89081 Germany; 2 Molecular Psychology, Department of Psychology, Humboldt-Universität zu Berlin, Berlin, Germany

**Keywords:** TikTok, Social media addiction, Personality, Neuroticism, Conscientiousness, Depression

## Abstract

**Background:**

We introduce a novel measure for assessing TikTok overuse, called the TikTok Use Disorder-Questionnaire (TTUD-Q). As part of ongoing investigations into the suitability of the World Health Organization’s (WHO) framework for diagnosing Gaming Disorder in the context of social media overuse, we developed this questionnaire by adapting the WHO framework, replacing the term “gaming” with “TikTok use”.

**Methods:**

In order to address this question, we investigated the psychometric properties of the newly designed TTUD-Q and assessed its associations with the BFI-10 (assessing the Big Five of Personality) and the PHQ-8 (assessing depressive tendencies).

**Results:**

In this study, involving a final sample of 378 participants, we observed that higher levels of neuroticism were linked to greater tendencies toward TikTok Use Disorder (TTUD). Furthermore, we identified that this association was mediated by depressive tendencies. Similar trends emerged when investigating the relationship between lower levels of conscientiousness and higher TTUD tendencies, with depressive tendencies once again serving as a mediator.

**Discussion:**

Our research sets the foundation for future studies that should delve deeper into examining individual differences in TTUD using the WHO framework originally designed for Gaming Disorder.

**Supplementary Information:**

The online version contains supplementary material available at 10.1186/s40359-024-01541-y.

## Introduction

Worldwide around five billion people use social media to connect, communicate and seek information [1]. Despite the evident advantages of utilizing social media, such as the augmentation of social capital [2], an ongoing controversy surrounds the potential adverse effects of its usage. Specifically, there is an observed tendency among certain users to escalate their usage of social media over time [3, 4], leading to a dependency that manifests symptoms reminiscent of those associated with substance or behavioral addiction. As social media platforms differ in their platform design (and therefore also likely in the user group they attract), it is highly relevant to investigate the effect of distinct platform usage on mental health [5, 6]. The Chinese TikTok platform operated by ByteDance, a social media platform centered around short video sharing, has indeed been somewhat overlooked in research [7]. This is particularly noticeable when juxtaposed with the extensive investigations conducted on Meta platforms. We advocate to also focus on TikTok, given that this platform has now amassed over one billion estimated users. While research on general social media overuse has garnered significant attention in recent years [8], we believe that a shift towards examining the overuse of specific social media platforms is crucial. This emphasis is warranted because social media platforms vary in design, suggesting potential differences in their addictive potentials [6]. Consequently, distinct platforms may exert varying influences on users, potentially resulting in diverse consequences for their mental health. Due to the relatively limited literature on TikTok use, especially when contrasted with studies on overuse of other social media platforms, the current study specifically concentrates on TikTok overuse.

Our study, aimed at comprehending TikTok (over-)use, is anchored in the I-PACE model, a theoretical framework delineating the processes involved in the development and perpetuation of addictive use in specific internet applications [9]. The acronym I-PACE stands for the *I*nteraction of *P*erson-*A*ffect-*C*ognition and *E*xecution variables, providing insights into the development of addictive behaviors in the online domain. Of particular importance for the present work is the P-variable of the I-PACE model comprising also the personality-category. Focusing on personality to better understand individual differences in TikTok (over-)use has been shown to be of potential relevance also in a recent work [10], but this study did focus on engagement with TikTok. Moreover, research also supports the significance of personality traits in understanding who is more to be enticed by the design of social media platforms [11]. A meta-analysis investigating the Big Five of Personality in the context of “Social Media Addiction” revealed that higher neuroticism and lower conscientiousness scores were notably associated with increased tendencies to overuse social media [12]. As a result, we hypothesize that this particular combination of personality traits would be linked with higher TikTok Use Disorder tendencies. It is important to emphasize, however, that overuse of social media is currently not recognized as an official diagnosis. This field remains a subject of ongoing and controversial discussion [13, 14]. On the other hand, it’s worth noting that Gaming Disorder and Gambling Disorder are both included in the current International Classification of Diseases-11 (ICD-11) issued by the World Health Organization (WHO) [15]. Therefore, these established disorders could potentially serve as a model for examining disordered social media use and may offer valuable insights into this emerging area of study. In order to test this, we adapted the Gaming Disorder Test items [16] which were originally designed to assess symptoms related to Gaming Disorder following the WHO’s Gaming Disorder framework. Gaming Disorder is described by several symptoms including loss of control, prioritizing gaming beyond other essential daily activities, continuing gaming despite negative consequences, and functional impairments. It is important to note that these behaviors/symptoms should be observed over an extended period, typically spanning 12 months, to be considered indicative of Gaming Disorder. In the present work, we replaced the term “gaming” with “TikTok use” in these items (the original wording of the items can be found at the Open Science Framework, see below). In line with the terminology used in the context of Gaming Disorder and Gambling Disorder, we refer to this condition as TikTok Use Disorder (TTUD), but explicitly mention that we do not intend to pathologize everyday life behavior [17]. We use this term to align with the terminology used in the study of excessive gaming and gambling in ICD-11. Additionally, we refer to TTUD tendencies because our study primarily focused on investigating a subclinical sample. It may be appropriate to incorporate symptoms from the WHO framework for diagnosing Gaming Disorder in the present study context, given the observed overlap between (Internet) Gaming Disorder and tendencies associated with Social Networks Use Disorder, as indicated by literature [18–20]. However, it is essential to recognize that these studies did not employ the WHO framework for Gaming Disorder; instead, they relied on older and non-officially recognized classification schemes.

Given that a substantial body of research suggests connections between the overuse of social media and negative emotionality [21, 22], our first objective was to investigate the link between TTUD and depressive tendencies. Although we only have cross-sectional data and cannot establish causality between the investigated variables, we hypothesized that the connections between higher neuroticism/lower conscientiousness and higher TTUD tendencies could be mediated by depressive tendencies. For instance, it has been previously reported that neuroticism is a risk factor for developing depression [23], and negative affect could potentially drive individuals to cope through the use of social media, such as TikTok (as discussed in a study framing internet overuse as a coping mechanism [24]), which, in turn, might lead to excessive or disordered usage. While alternative variable configurations are certainly conceivable, our model was designed in line with some existing literature (see above). Please note, that data underlying this work are available at the Open Science Framework (https://osf.io/nf2u5/), and we encourage interested researchers to explore other alternative models.

## Methods

### Participants

For the present study, we included a total of 383 participants from a much larger initial sample of 7111 individuals. Only this smaller subset of participants answered affirmatively to questions regarding their use of both social media and TikTok and was at least 18 years of age, and thus met the criteria for inclusion in our study. Social media and TikTok use were assessed with two items by simply asking the study participants if they use social media (yes/no) and if they use TikTok (yes/no). Additionally, there were four participants who identified as a third gender. Due to the limited number of participants in this group, they were (unfortunately) excluded from the analysis, resulting in a sample of 379 participants. Subsequently, after conducting a careful analysis to identify and exclude instances of careless responding (checking for monotonous answer patterns in the BFI-10, including its inverse items), the sample size was slightly reduced to a total of 378 participants. The final sample of study participants comprised 124 males (32,80%) and 254 females (67,20%) with a mean-age of 40.92 years (SD = 16.10).

Please note that the present work was advertised to be a study on cognitive failure and participants were provided with feedback on their cognitive failure scores (in an anonymous way; we assessed for another study the cognitive failure questionnaire to assess individual differences in everyday cognitive lapses; see [25]). Hence the framing of the study could have drawn a non-typical sample for TikTok users. This indeed this seems to be the case, because TikTok users are usually characterized by being of younger age and we here investigate an older TikTok user sample, which nevertheless can provide interesting insights. The questionnaire of relevance to be studied in the present context are presented in the following. Please note, that none of these questionnaire data (BFI-10, PHQ-8, TTUD-Q) from this sample has been presented elsewhere. The study was approved by the local ethics committee at HU Berlin, Berlin, Germany.

### Questionnaires

#### TikTok Use Disorder-Questionnaire (TTUD-Q)

The TTUD-Q represents an adaption of the already existing GDT consisting of four items being answered on a five-point Likert scale ranging from 1 = never to 5 = very often [16, 26]. The German version was adapted [26]. Items assess a loss of control over TikTok use, the priorization of TikTok use over other daily activities, continued use of TikTok despite the experience of negative consequences, as well as functional impairment due to TikTok use over a time course of twelve month. Higher scores indicate higher TTUD tendencies. Internal consistencies are in the satisfying range (complete scale: α = 0.788; factor 1: α = 0.835; factor 2: α = 0.649). CFA modeling revealed that the model in our data exhibited a satisfactory fit: We created a latent variable for items 1 and 2 and another latent variable for items 3 and 4, resulting in the following model fit statistics: Chi2 = 1.67, df = 1, *p* = 0.196; CFI = 0.999, TLI = 0.993, RMSEA = 0.0420 (90% CI: lower 0.00, upper 0.151). As a result, we were able to present findings related to subscales called “loss of control/priority (I)” and “impairment (II)”. Please keep in mind that the German version of the questionnaire was used in this study, and the psychometric properties of the English version still need to be established. The exact wording of the items can be found in a separate file at the Open Science Framework (https://osf.io/nf2u5/; the journal policy does not allow other language than English in the Supplementary Material).

#### Patient Health Questionnaire-8 (PHQ-8)

Furthermore, a German version of the PHQ-8 (see English version here [27]) was employed, which comprises eight items. Items assess impairment due to experienced symptoms of depression such as having no energy, less appetite, difficulties to concentrate, feelings of hopelessness and few interest in activities in the last two weeks. The suicide item was excluded from the PHQ-9 version (citation above for PHQ-9) to come up with the PHQ-8-version in the present work. Items were answered with a four-answer format ranging from 1 = having not felt the respective symptom in the last two weeks to 4 = felt the respective symptom nearly every day. Higher scores indicate higher depressive tendencies and internal consistencies again were satisfying: α = 0.835.

#### Big Five Inventory-10 (BFI-10)

Finally, the German version of the BFI-10 was administered as a brief measure to assess the Big Five of Personality [28]. The ten items are answered with a five-answer format ranging from 1 = does not apply at all to 5 = applies very much. Higher scores mean higher openness to experience, higher conscientiousness, higher extraversion, higher agreeableness and higher neuroticism (each assessed with two items, please note that five items need to be reversed before they can be summed). In particular, conscientiousness and neuroticism were of interest for the present study.

### Statistical analysis

We used the Jamovi package 2.3.18.0 for statistical analyses. Descriptive statistics are presented both for the total sample and the male/female subsamples. Gender differences in the relevant psychological dimensions are presented both with T-Tests and Mann–Whitney-U-Tests (to check for stability of findings). The TTUD-Q variable is skewedly distributed, therefore we present correlation tables with both parametric and non-parametric coefficients (Pearson and Spearman correlations). Mediation models were calculated with personality (either neuroticism or conscientiousness) as predictor variable, TTUD-Q as outcome variable and PHQ-8 as the mediation variable. The mediation model was carried out with the “medmod” module within the Jamovi package. The mediation model was deemed to be appropriate, as parametric and non-parametric correlations resulted in comparable findings. Nevertheless, we hint to the fact that the TTUD scores are strongly deviating from normal distribution (and the data is available to run further models). Please note that such mediation model does not imply causality.

## Results

Table 1 presents the descriptive statistics for all variables. Notably, the TTUD-Q scores in the sample appear to be concentrated in the lower range. Tables 2 and 3 reveal that, on average, females tend to have higher scores on all variables, except for age, where the opposite trend is observed. The differences were in particular pronounced for neuroticism, TTUD-Q, PHQ-8 and age (see also effect sizes in Table 3). In Table 4, correlations between variables are presented. In alignment with our hypothesis, there are positive associations between neuroticism/PHQ-8, and TTUD-Q (neuroticism and TTUD-Q: *r* = 0.196, *p* < 0.001; PHQ-8 and TTUD-Q: *r* = 0.309, *p* < 0.001; please note that Spearman correlations are slightly smaller). Furthermore, there is an inverse association between conscientiousness and both PHQ-8/TTUD-Q (r = -0.304, *p* < .001; *r* = -0.185, *p* < 0.001; here a bit higher with Spearman correlations). Despite some small differences in effect sizes, the observed findings are consistent across parametric and non-parametric correlations. Additionally, several significant age-related associations are evident, particularly a moderate negative association between TTUD-Q and age (*r* = -0.445, *p* < 0.001). Thus, age and gender are crucial variables that should be considered in the analysis.

**Table 1:** Descriptives statistics for the complete sample

									**Skewness**		**Kurtosis**	
	**N**	**Missing**	**Mean**	**Median**	**SD**	**Range**	**Minimum**	**Maximum**	**Skewness**	**SE**	**Kurtosis**	**SE**
TikTokUD	378	0	6.20	5.00	2.830	14.00	4.00	18.00	1.37605	0.125	1.1900	0.250
TikTokUD_Factor1	378	0	3.70	3.00	2.066	8.00	2.00	10.00	1.06051	0.125	0.0541	0.250
TikTokUD_Factor2	378	0	2.50	2.00	1.081	7.00	2.00	9.00	2.63548	0.125	7.6633	0.250
PHQ-8	378	0	16.78	16.00	5.017	24.00	8.00	32.00	0.54046	0.125	-0.2874	0.250
BFI_Extraversion	378	0	3.07	3.00	1.064	4.00	1.00	5.00	0.04194	0.125	-0.8872	0.250
BFI_Neuroticism	378	0	3.31	3.50	1.024	4.00	1.00	5.00	-0.18842	0.125	-0.7654	0.250
BFI_Openness	378	0	3.75	4.00	0.948	4.00	1.00	5.00	-0.59735	0.125	-0.3270	0.250
BFI_Conscientiousness	378	0	3.17	3.00	0.883	4.00	1.00	5.00	0.00583	0.125	-0.4377	0.250
BFI_Agreeableness	378	0	3.23	3.00	0.796	4.00	1.00	5.00	-0.10848	0.125	-0.3037	0.250
Age	378	0	40.92	40.00	16.101	67.00	18.00	85.00	0.36823	0.125	-0.6653	0.250

*TikTokUD* TikTok Use Disorder (as assessed with the TTUD-Q), *TikTokUD_Factor1* Loss of Control/Priority, *TikTokUD_Factor2* Impairment, *PHQ-8* Patient Health Questionnaire

**Table 2:** Descriptives statistics for the male and female subsample (1 = males, 2 = females)

										**Skewness**		**Kurtosis**	
	**Gender**	**N**	**Missing**	**Mean**	**Median**	**SD**	**Range**	**Minimum**	**Maximum**	**Skewness**	**SE**	**Kurtosis**	**SE**
TikTokUD	1	124	0	5.53	4.00	2.218	10.00	4.00	14.00	1.77270	0.217	2.93516	0.431
	2	254	0	6.53	5.00	3.036	14.00	4.00	18.00	1.18372	0.153	0.56237	0.304
TikTokUD_Factor1	1	124	0	3.20	2.00	1.687	8.00	2.00	10.00	1.42276	0.217	1.65712	0.431
	2	254	0	3.95	3.00	2.189	8.00	2.00	10.00	0.88387	0.153	-0.44222	0.304
TikTokUD_Factor2	1	124	0	2.33	2.00	0.881	5.00	2.00	7.00	3.21382	0.217	10.95134	0.431
	2	254	0	2.58	2.00	1.159	7.00	2.00	9.00	2.42401	0.153	6.52386	0.304
PHQ-8	1	124	0	15.79	15.00	4.970	24.00	8.00	32.00	0.71207	0.217	0.18141	0.431
	2	254	0	17.27	16.00	4.978	24.00	8.00	32.00	0.48581	0.153	-0.42224	0.304
BFI_Extraversion	1	124	0	2.91	3.00	0.966	4.00	1.00	5.00	0.11594	0.217	-0.71107	0.431
	2	254	0	3.14	3.00	1.102	4.00	1.00	5.00	-0.03031	0.153	-0.95822	0.304
BFI_Neuroticism	1	124	0	2.89	3.00	0.989	4.00	1.00	5.00	0.04449	0.217	-0.86692	0.431
	2	254	0	3.51	3.50	0.979	4.00	1.00	5.00	-0.30437	0.153	-0.61683	0.304
BFI_Openness	1	124	0	3.58	3.50	0.939	3.50	1.50	5.00	-0.24961	0.217	-0.58770	0.431
	2	254	0	3.84	4.00	0.943	4.00	1.00	5.00	-0.78869	0.153	-0.00945	0.304
BFI_Conscientiousness	1	124	0	3.03	3.00	0.832	4.00	1.00	5.00	-0.08017	0.217	-0.24630	0.431
	2	254	0	3.24	3.00	0.900	4.00	1.00	5.00	0.00586	0.153	-0.54148	0.304
BFI_Agreeableness	1	124	0	3.08	3.00	0.771	3.50	1.50	5.00	-0.01842	0.217	-0.35726	0.431
	2	254	0	3.31	3.50	0.799	4.00	1.00	5.00	-0.16985	0.153	-0.22448	0.304
Age	1	124	0	46.88	47.00	16.953	63.00	18.00	81.00	-0.04026	0.217	-0.85327	0.431
	2	254	0	38.02	37.00	14.857	67.00	18.00	85.00	0.52932	0.153	-0.34145	0.304

**Table 3:** Independent Samples T-Tests/Mann-Whitney U tests investigating gender effects in the realm of the variables presented

		**Statistic**	**df**	***p***		**Effect Size**
TikTokUD	Student's t	-3.25^a^	376	0.001	Cohen's d	-0.356
	Welch's t	-3.61	321	< .001	Cohen's d	-0.374
	Mann-Whitney U	12904		0.003	Rank biserial correlation	0.181
TikTokUD_Factor1	Student's t	-3.35^a^	376	< .001	Cohen's d	-0.367
	Welch's t	-3.65	307	< .001	Cohen's d	-0.382
	Mann-Whitney U	12638		0.001	Rank biserial correlation	0.197
TikTokUD_Factor2	Student's t	-2.10^a^	376	0.036	Cohen's d	-0.231
	Welch's t	-2.31	311	0.022	Cohen's d	-0.241
	Mann-Whitney U	14031		0.022	Rank biserial correlation	0.109
PHQ-8	Student's t	-2.71	376	0.007	Cohen's d	-0.297
	Welch's t	-2.71	244	0.007	Cohen's d	-0.297
	Mann-Whitney U	12968		0.005	Rank biserial correlation	0.177
BFI_Extraversion	Student's t	-2.04^a^	376	0.042	Cohen's d	-0.223
	Welch's t	-2.13	275	0.034	Cohen's d	-0.228
	Mann-Whitney U	13778		0.046	Rank biserial correlation	0.125
BFI_Neuroticism	Student's t	-5.78	376	< .001	Cohen's d	-0.634
	Welch's t	-5.76	242	< .001	Cohen's d	-0.633
	Mann-Whitney U	10407		< .001	Rank biserial correlation	0.339
BFI_Openness	Student's t	-2.44	376	0.015	Cohen's d	-0.268
	Welch's t	-2.45	245	0.015	Cohen's d	-0.268
	Mann-Whitney U	13059		0.006	Rank biserial correlation	0.171
BFI_Conscientiousness	Student's t	-2.18^a^	376	0.030	Cohen's d	-0.239
	Welch's t	-2.24	262	0.026	Cohen's d	-0.242
	Mann-Whitney U	13858		0.054	Rank biserial correlation	0.120
BFI_Agreeableness	Student's t	-2.59	376	0.010	Cohen's d	-0.284
	Welch's t	-2.62	252	0.009	Cohen's d	-0.286
	Mann-Whitney U	13232		0.010	Rank biserial correlation	0.160
Age	Student's t	5.20	376	< .001	Cohen's d	0.569
	Welch's t	4.96	218	< .001	Cohen's d	0.556
	Mann-Whitney U	10884		< .001	Rank biserial correlation	0.309

Note. H_a _μ_1_ ≠ μ_2_

^a^Levene's test is significant (*p* < .05), suggesting a violation of the assumption of equal variances

**Table 4:** Correlation matrix

		**TikTokUD**	**TikTokUD_Factor1**	**TikTokUD_Factor2**	**PHQ-8**	**BFI_E**	**BFI_N**	**BFI_O**	**BFI_C**	**BFI_A**	**Age**
TikTokUD	Pearson’s r	—									
	*p*-value	—									
	Spearman’s rho	—									
	*p*-value	—									
TikTokUD_Factor1	Pearson’s r	0.950^***^	—								
	*p*-value	< .001	—								
	Spearman’s rho	0.972^***^	—								
	*p*-value	< .001	—								
TikTokUD_Factor2	Pearson’s r	0.802^***^	0.576^***^	—							
	*p*-value	< .001	< .001	—							
	Spearman’s rho	0.689^***^	0.551^***^	—							
	*p*-value	< .001	< .001	—							
PHQ-8	Pearson’s r	0.309^***^	0.269^***^	0.294^***^	—						
	*p*-value	< .001	< .001	< .001	—						
	Spearman’s rho	0.254^***^	0.233^***^	0.256^***^	—						
	*p*-value	< .001	< .001	< .001	—						
BFI_Extraversion	Pearson’s r	-0.011	0.007	-0.043	-0.139^**^	—					
	*p*-value	0.831	0.890	0.410	0.007	—					
	Spearman’s rho	0.004	0.009	-0.032	-0.140^**^	—					
	*p*-value	0.943	0.860	0.540	0.006	—					
BFI_Neuroticism	Pearson’s r	0.196^***^	0.185^***^	0.159^**^	0.400^***^	-0.159^**^	—				
	*p*-value	< .001	< .001	0.002	< .001	0.002	—				
	Spearman’s rho	0.150^**^	0.163^**^	0.162^**^	0.423^***^	-0.156^**^	—				
	*p*-value	0.004	0.002	0.002	< .001	0.002	—				
BFI_Openness	Pearson’s r	-0.045	-0.056	-0.011	0.005	0.140^**^	0.051	—			
	*p*-value	0.380	0.275	0.831	0.915	0.006	0.323	—			
	Spearman’s rho	-0.024	-0.016	-0.013	0.012	0.143^**^	0.066	—			
	*p*-value	0.636	0.754	0.808	0.822	0.005	0.200	—			
BFI_Conscientiousness	Pearson’s r	-0.185^***^	-0.188^***^	-0.126^*^	-0.304^***^	0.109^*^	-0.050	-0.059	—		
	*p*-value	< .001	< .001	0.014	< .001	0.033	0.332	0.249	—		
	Spearman’s rho	-0.206^***^	-0.199^***^	-0.149^**^	-0.306^***^	0.103^*^	-0.061	-0.069	—		
	*p*-value	< .001	< .001	0.004	< .001	0.045	0.235	0.183	—		
BFI_Agreeableness	Pearson’s r	-0.036	-0.013	-0.069	-0.045	0.106^*^	0.026	0.089	-0.012	—	
	*p*-value	0.485	0.801	0.178	0.378	0.039	0.613	0.084	0.823	—	
	Spearman’s rho	-0.022	-0.019	-0.067	-0.022	0.097	0.010	0.099	-0.014	—	
	*p*-value	0.667	0.718	0.192	0.672	0.059	0.849	0.055	0.791	—	
Age	Pearson’s r	-0.445^***^	-0.462^***^	-0.280^***^	-0.198^***^	-0.034	-0.133^**^	0.013	0.207^***^	-0.002	—
	*p*-value	< .001	< .001	< .001	< .001	0.510	0.010	0.796	< .001	0.972	—
	Spearman’s rho	-0.492^***^	-0.495^***^	-0.324^***^	-0.197^***^	-0.034	-0.136^**^	-0.026	0.204^***^	-0.009	—
	*p*-value	< .001	< .001	< .001	< .001	0.506	0.008	0.608	< .001	0.864	—

^*^p < .05, ^**^p < .01, ^***^p < .001

Mediation analysis, as shown in Tables 5 and 6, supports the idea of full mediation for the neuroticism-depression-TTUD link and a near full mediation model for the conscientiousness-depression-TTUD link. The Supplementary Material (see Figure SF1, Tables S1 and S2) further demonstrates that comparable results are obtained when age and gender are included in the model.

**Table 5:** Mediation model with the variables Neuroticism (predictor), PHQ-8 (mediator) and TTUD-Q (outcome)

				**95% C.I. (a)**				
**Type**	**Effect**	**Estimate**	**SE**	**Lower**	**Upper**	**β**	**z**	**p**
Indirect	BFI_Neuroticism ⇒ PHQ-8 ⇒ TikTokUD	0.303	0.0688	0.1686	0.438	0.1098	4.41	< .001
Component	BFI_Neuroticism ⇒ PHQ-8	1.957	0.2310	1.5047	2.410	0.3996	8.47	< .001
	PHQ-8 ⇒ TikTokUD	0.155	0.0300	0.0962	0.214	0.2748	5.17	< .001
Direct	BFI_Neuroticism ⇒ TikTokUD	0.237	0.1469	-0.0507	0.525	0.0859	1.61	0.106
Total	BFI_Neuroticism ⇒ TikTokUD	0.541	0.1395	0.2671	0.814	0.1957	3.87	< .001

Confidence intervals computed with method: Standard (Delta method)

Betas are completely standardized effect sizes; BFI_Neuroticism = personality trait; PHQ-8 = depressive tendencies; TTUD = TikTok Use Disorder tendencies

**Table 6:** Mediation model with the variables Conscientiousness (predictor), PHQ-8 (mediator) and TTUD-Q (outcome)

				**95% C.I. (a)**				
**Type**	**Effect**	**Estimate**	**SE**	**Lower**	**Upper**	**β**	**z**	**p**
Indirect	BFI_Conscientiousness ⇒ PHQ-8 ⇒ TikTokUD	-0.271	0.0663	-0.401	-0.14143	-0.0846	-4.09	< .001
Component	BFI_Conscientiousness ⇒ PHQ-8	-1.727	0.2786	-2.273	-1.18086	-0.3038	-6.20	< .001
	PHQ-8 ⇒ TikTokUD	0.157	0.0288	0.101	0.21357	0.2786	5.45	< .001
Direct	BFI_Conscientiousness ⇒ TikTokUD	-0.322	0.1638	-0.643	-0.00148	-0.1006	-1.97	0.049
Total	BFI_Conscientiousness ⇒ TikTokUD	-0.594	0.1623	-0.912	-0.27572	-0.1852	-3.66	< .001

Confidence intervals computed with method: Standard (Delta method)

Betas are completely standardized effect sizes; BFI_Conscientiousness = personality trait; PHQ-8 = depressive tendencies; TTUD = TikTok Use Disorder tendencies

## Discussion

The present study aimed to explore a new measure of TikTok Use Disorder (called TTUD-Q), which was developed within the framework of the WHO’s Gaming Disorder guidelines. The questionnaire exhibited satisfactory psychometric properties, and notably, it confirmed the anticipated associations with neuroticism, conscientiousness, and depressive tendencies. This positions the current TTUD-Q as a promising avenue for future research aimed at gaining a deeper understanding of TikTok overuse. However, it is crucial to recognize that this study serves as an initial exploration, marking the commencement of efforts to ascertain whether the WHO framework for Gaming Disorder is transferable to the investigation of social media, specifically TikTok overuse. The preliminary findings, evaluated from both psychometric perspectives and external validation through associations with personality and depression, appear encouraging. It is essential to note, though, that the present work assessed the German version of the TTUD-Q, and further testing is imperative for other languages, including the items presented in English. The mediation model further reinforced the notion that higher neuroticism could lead to increased levels of depression, which, in turn, might result in heightened TTUD-tendencies, potentially as a result from coping mechanisms for experienced negative affect [24]. As a result, individuals with elevated neuroticism scores may encounter heightened negative emotionality in their daily lives, prompting the use of social media—specifically TikTok—as a means of escapism to alleviate these negative emotions. If this coping strategy becomes predominant for addressing everyday challenges, it could potentially lead not only to the development of addictive behaviors related to a social media platform like TikTok but also hinder the resolution of personal life issues. Additionally, it was observed that lower conscientiousness was associated with higher TTUD tendencies, with depressive tendencies serving as a mediator. The finding regarding conscientiousness should be taken into account alongside the findings on neuroticism. When individuals experience negative affect and have reduced self-regulation abilities, they may be more prone to being drawn to a social media platform such as TikTok as a way to escape the challenges of everyday life. Of note, recent research underscores that individuals with depression often exhibit higher neuroticism and lower conscientiousness [29], which aligns well with our findings. Overall, the study suggests that high neuroticism and low conscientiousness may represent vulnerability factors for developing TTUD tendencies, a notion that also resonates with a meta-analysis on personality and social media overuse [12]. The role of negative affect as a potential mediator aligns with the I-PACE model [9]).

However, it’s crucial to acknowledge the study’s limitations. Firstly, it is of a cross-sectional nature, making it impossible to establish causal relationships between the variables. Secondly, the study relied on a convenience sample, which raises questions about the generalizability of the results, in particular to younger study populations. Thirdly, the study may be influenced by social desirability biases, memory biases, and the specific framework utilized, as discussed in the methods section.

Despite these limitations, the findings are consistent with our expectations. Higher neuroticism and lower conscientiousness appear to be important factors in understanding TTUD tendencies, as assessed using the WHO framework designed for Gaming Disorder. Future research should explore the applicability of the WHO framework to social media overuse, not only for TikTok but also for other platforms. The current study serves as a modest contribution to unraveling the complexities of social media overuse, particularly with a focus on the relatively less-explored TikTok platform. The familiar personality profile—characterized by high neuroticism and low conscientiousness—appears to be a vulnerability factor for heightened tendencies towards TTUD, aligning with research on non-specific Social Networks Use Disorder tendencies (for more on this terminology see Rumpf et al., 2021) [30]. To gain a more nuanced understanding of social media overuse, especially on platforms like TikTok, further studies are imperative. Additionally, these investigations should explore whether the broader category of Social Networks Use Disorder warrants consideration as a distinct disorder in future editions of the ICD. Future studies ought to extend beyond subclinical study populations, such as the present one, to assess the framework’s effectiveness in differentiating between healthy individuals and those with clear pathological use of social media.

The current study suggests that the framework proposed by the WHO for diagnosing Gaming Disorder may be suitable for diagnosing SNUD (here investigating TTUD tendencies). However, we exercise caution in not overinterpreting our data at this point. As we conclude this work, we encourage other researchers to further investigate and scrutinize the WHO framework for Gaming Disorder in the context of social media overuse. This exploration should extend to the examination of specific platforms, considering their distinct designs and potential to instigate addictive behaviors.

### Supplementary Information

**Additional file 1. **Supplementary material accompanying the present paper.

## Data Availability

The data are available at the Open Science Framework: https://osf.io/nf2u5/
