# Supplementary material for: Depressive inclinations mediate the association between personality (neuroticism/conscientiousness) and TikTok Use Disorder tendencies
Source: BMC Psychol. 2024 Feb 17;12:81. doi: 10.1186/s40359-024-01541-y (PMC10873925; doi:10.1186/s40359-024-01541-y)
Supplement: Supplementary file 1 — Additional file 1. Supplementary material accompanying the present paper. [file 40359_2024_1541_MOESM1_ESM.docx]

Supplementary material accompanying paper “Depressive inclinations mediate the association between personality (neuroticism/conscientiousness) and TikTok Use Disorder tendencies”


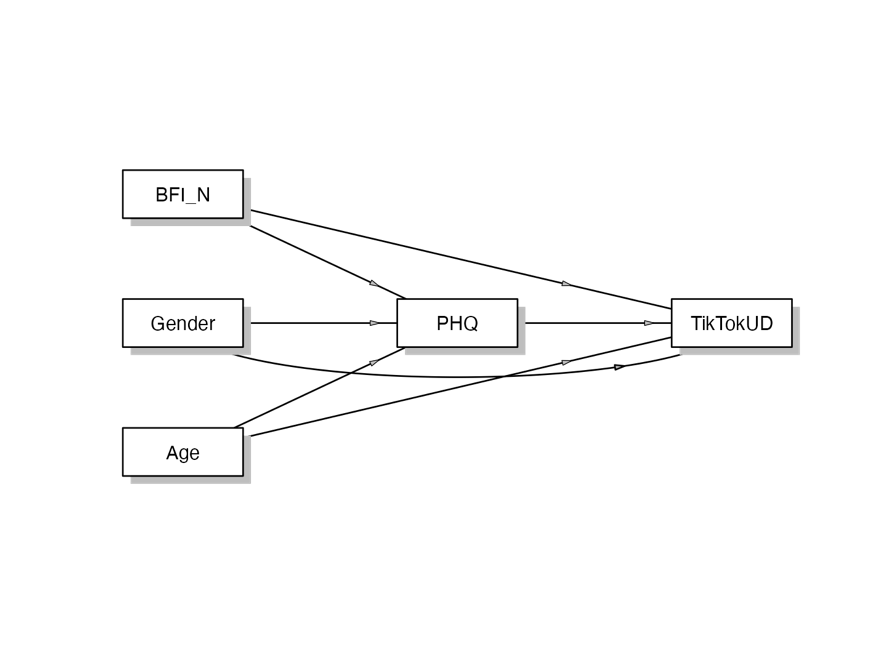


SF 1: Depicts the model to be tested in the supplementary material including age and gender, please note that in one model neuroticism (BFI_N) was tested (as shown) and in another model conscientiousness (not depicted); PHQ: Patient Health Questionnaire (PHQ-8), TikTokUD: TikTok Use Disorder

| ST1: Mediation model 1 including age and gender variables | | | | | | | | | | | | | | | | | |
| --- | --- | --- | --- | --- | --- | --- | --- | --- | --- | --- | --- | --- | --- | --- | --- | --- | --- |
|  | | | | | | | | **95% C.I. (a)** | | | |  | | | | | |
| **Type** | | **Effect** | | **Estimate** | | **SE** | | **Lower** | | **Upper** | | **β** | | **z** | | **p** | |
| Indirect |  | BFI_N ⇒ PHQ-8 ⇒ TikTokUD |  | 0.21825 |  | 0.05891 |  | 0.10278 |  | 0.33371 |  | 0.07899 |  | 3.705 |  | < .001 |  |
|  |  | Gender ⇒ PHQ-8 ⇒ TikTokUD |  | -0.01215 |  | 0.06215 |  | -0.13396 |  | 0.10966 |  | -0.00202 |  | -0.195 |  | 0.845 |  |
|  |  | Age ⇒ PHQ-8 ⇒ TikTokUD |  | -0.00545 |  | 0.00218 |  | -0.00973 |  | -0.00118 |  | -0.03104 |  | -2.501 |  | 0.012 |  |
| Component |  | BFI_N ⇒ PHQ-8 |  | 1.87347 |  | 0.23839 |  | 1.40623 |  | 2.34071 |  | 0.38244 |  | 7.859 |  | < .001 |  |
|  |  | PHQ-8 ⇒ TikTokUD |  | 0.11649 |  | 0.02773 |  | 0.06214 |  | 0.17084 |  | 0.20655 |  | 4.201 |  | < .001 |  |
|  |  | Gender ⇒ PHQ-8 |  | -0.10430 |  | 0.53292 |  | -1.14881 |  | 0.94021 |  | -0.00977 |  | -0.196 |  | 0.845 |  |
|  |  | Age ⇒ PHQ-8 |  | -0.04682 |  | 0.01504 |  | -0.07631 |  | -0.01734 |  | -0.15026 |  | -3.112 |  | 0.002 |  |
| Direct |  | BFI_N ⇒ TikTokUD |  | 0.15382 |  | 0.13863 |  | -0.11789 |  | 0.42554 |  | 0.05567 |  | 1.110 |  | 0.267 |  |
|  |  | Gender ⇒ TikTokUD |  | 0.11828 |  | 0.28734 |  | -0.44490 |  | 0.68145 |  | 0.01965 |  | 0.412 |  | 0.681 |  |
|  |  | Age ⇒ TikTokUD |  | -0.06872 |  | 0.00821 |  | -0.08482 |  | -0.05263 |  | -0.39105 |  | -8.367 |  | < .001 |  |
| Total |  | BFI_N ⇒ TikTokUD |  | 0.37207 |  | 0.13167 |  | 0.11400 |  | 0.63014 |  | 0.13467 |  | 2.826 |  | 0.005 |  |
|  |  | Gender ⇒ TikTokUD |  | 0.10613 |  | 0.29435 |  | -0.47078 |  | 0.68303 |  | 0.01763 |  | 0.361 |  | 0.718 |  |
|  |  | Age ⇒ TikTokUD |  | -0.07418 |  | 0.00831 |  | -0.09046 |  | -0.05789 |  | -0.42209 |  | -8.928 |  | < .001 |  |
| Note. Confidence intervals computed with method: Standard (Delta method) | | | | | | | | | | | | | | | | | |
| Note. Betas are completely standardized effect sizes  BFI_N: Neuroticism, PHQ-8: Patient Health Questionnaire, TikTokUD: TikTok Use Disorder | | | | | | | | | | | | | | | | | |
|  | | | | | | | | | | | | | | | | | |

| ST2: Mediation model 2 including age and gender | | | | | | | | | | | | | | | | | |
| --- | --- | --- | --- | --- | --- | --- | --- | --- | --- | --- | --- | --- | --- | --- | --- | --- | --- |
|  | | | | | | | | **95% C.I. (a)** | | | |  | | | | | |
| **Type** | | **Effect** | | **Estimate** | | **SE** | | **Lower** | | **Upper** | | **β** | | **z** | | **p** | |
| Indirect |  | Gender ⇒ PHQ-8 ⇒ TikTokUD |  | 0.18819 |  | 0.07726 |  | 0.03677 |  | 0.3396 |  | 0.0313 |  | 2.436 |  | 0.015 |  |
|  |  | Age ⇒ PHQ-8 ⇒ TikTokUD |  | -0.00370 |  | 0.00209 |  | -0.00780 |  | 4.01e-4 |  | -0.0210 |  | -1.768 |  | 0.077 |  |
|  |  | BFI_C ⇒ PHQ-8 ⇒ TikTokUD |  | -0.20520 |  | 0.05715 |  | -0.31722 |  | -0.0932 |  | -0.0640 |  | -3.590 |  | < .001 |  |
| Component |  | Gender ⇒ PHQ-8 |  | 1.56291 |  | 0.53808 |  | 0.50830 |  | 2.6175 |  | 0.1464 |  | 2.905 |  | 0.004 |  |
|  |  | PHQ-8 ⇒ TikTokUD |  | 0.12041 |  | 0.02693 |  | 0.06764 |  | 0.1732 |  | 0.2135 |  | 4.472 |  | < .001 |  |
|  |  | Age ⇒ PHQ-8 |  | -0.03072 |  | 0.01596 |  | -0.06200 |  | 5.52e-4 |  | -0.0986 |  | -1.925 |  | 0.054 |  |
|  |  | BFI_C ⇒ PHQ-8 |  | -1.70417 |  | 0.28296 |  | -2.25876 |  | -1.1496 |  | -0.2998 |  | -6.023 |  | < .001 |  |
| Direct |  | Gender ⇒ TikTokUD |  | 0.25399 |  | 0.28482 |  | -0.30424 |  | 0.8122 |  | 0.0422 |  | 0.892 |  | 0.373 |  |
|  |  | Age ⇒ TikTokUD |  | -0.06708 |  | 0.00839 |  | -0.08353 |  | -0.0506 |  | -0.3817 |  | -7.991 |  | < .001 |  |
|  |  | BFI_C ⇒ TikTokUD |  | -0.14813 |  | 0.15508 |  | -0.45208 |  | 0.1558 |  | -0.0462 |  | -0.955 |  | 0.339 |  |
| Total |  | Gender ⇒ TikTokUD |  | 0.44219 |  | 0.28943 |  | -0.12508 |  | 1.0095 |  | 0.0735 |  | 1.528 |  | 0.127 |  |
|  |  | Age ⇒ TikTokUD |  | -0.07078 |  | 0.00858 |  | -0.08760 |  | -0.0540 |  | -0.4027 |  | -8.246 |  | < .001 |  |
|  |  | BFI_C ⇒ TikTokUD |  | -0.35333 |  | 0.15220 |  | -0.65164 |  | -0.0550 |  | -0.1102 |  | -2.321 |  | 0.020 |  |
| Note. Confidence intervals computed with method: Standard (Delta method) | | | | | | | | | | | | | | | | | |
| Note. Betas are completely standardized effect sizes  BFI_C: Conscientiousness, PHQ-8: Patient Health Questionnaire, TikTokUD: TikTok Use Disorder | | | | | | | | | | | | | | | | | |
|  | | | | | | | | | | | | | | | | | |
